# Connecting Exosomes and Connexins

**DOI:** 10.3390/cancers11040476

**Published:** 2019-04-04

**Authors:** Joanna Gemel, John Kilkus, Glyn Dawson, Eric C. Beyer

**Affiliations:** Department of Pediatrics, University of Chicago, Chicago, IL 60637, USA; jgemel@peds.bsd.uchicago.edu (J.G.); 34kilk@gmail.com (J.K.); dawg@uchicago.edu (G.D.)

**Keywords:** connexin, extracellular vesicle, exosome, gap junction, intercellular communication, endothelial cell

## Abstract

Intercellular communication is accomplished by passage of ions and small molecules through gap junction channels in directly contacting cells or by secretion and response to transmitters, hormones and extracellular vesicles in cells that are distant from each other. Recent studies have suggested that there may be overlap of these processes; specifically, small extracellular vesicles may contain subunit gap junction proteins, connexins. We isolated and analyzed extracellular vesicles secreted by cultured microvascular endothelial cells. These vesicles had a diameter of ~120 nm. They contained four exosomal proteins (flotillin-1, CD63, CD81 and Alix) and the gap junction protein, connexin43. They did not contain an endoplasmic reticulum protein (Grp94) or an adherens junction protein (VE-cadherin). Secretion of vesicles was increased by treatment of the cells with staurosporine. Our data confirm that the gap junction protein, connexin43, can be secreted in vesicles with the properties of exosomes. Although the role of vesicular connexin is not clearly known, we speculate that it might participate in docking/fusion of the exosomes with the recipient cell, transmission of vesicular contents, or cellular signaling.

## 1. Introduction

Direct communication between cells is achieved by the passage of ions and small molecules through the channels contained within gap junctions. This process is critical for the function and survival of all multicellular tissues and organisms. Its disruption is associated with a wide variety of different diseases, including cancers.

In vertebrates, gap junction channels are formed by members of a family of subunit polypeptides called connexins (Cx). All of the connexins have substantial sequence and structural similarities; 21 different human connexin genes have been identified [1,2]. In addition to allowing intercellular communication, the connexins can form “hemi-channels” connecting the cytoplasm and extracellular space, and they can support cell adhesion and associations with other proteins at the plasma membrane. 

Cells may also achieve communication with other cells (even at a great distance) by the secretion of vesicles [3,4]. Exosomes are a subset of extracellular vesicles that have been defined based on their size, biogenesis, and contents (reviewed in [5,6,7]). Exosomes are formed inside endosomal Multivesicular Bodies by inward budding of membranes. When Multivesicular Bodies fuse with the plasma membrane, they release these intraluminal vesicles as exosomes. Recently, it has been postulated that lysosomal exocytosis may also be a source of exosomes [8]. Exosomes contain lipids, proteins, microRNA (miRNA), and messenger RNA (mRNA). If these components are taken up by target cells, the proteins could affect cellular signaling, and the miRNAs and mRNAs could modify gene transcription and translation. Exosome mediated signaling between cells may contribute to normal functions of organisms and tissues and to abnormal ones such as cancer (reviewed in [9,10]).

Recent publications have suggested functional links between gap junctions, connexins, and exosomes. Brink and colleagues [11] suggested that both gap junction channels and extracellular vesicles could facilitate the exchange of small nucleic acids between cells; the gap junctions would allow specific and direct exchange between adjacent cells, while vesicles would allow exchange that might be less specific, but accomplished over greater distances. Several proteomic studies have suggested the presence of connexins (including Cx32, Cx43, and Cx45) in extracellular vesicles (reviewed in [12]). Soares et al. [13] demonstrated that several cell types secreted vesicles containing Cx43, and they proposed critical roles for this protein in interactions with the target cells. The potential interaction of connexins and integrins in exosomes was recently reviewed [14].

We are particularly interested in signaling and cellular interactions involving cells of the blood vessel wall. Therefore, we performed studies to test whether cultured endothelial cells secret vesicles with properties consistent with exosomes and whether these extracellular vesicles contain a connexin that forms gap junctions between the cells.

## 2. Results

### 2.1. Cultured hCMEC/D3 Endothelial Cells Have Intercellular Junctions Containing Cx43 and VE-cadherin

**Figure 1:**
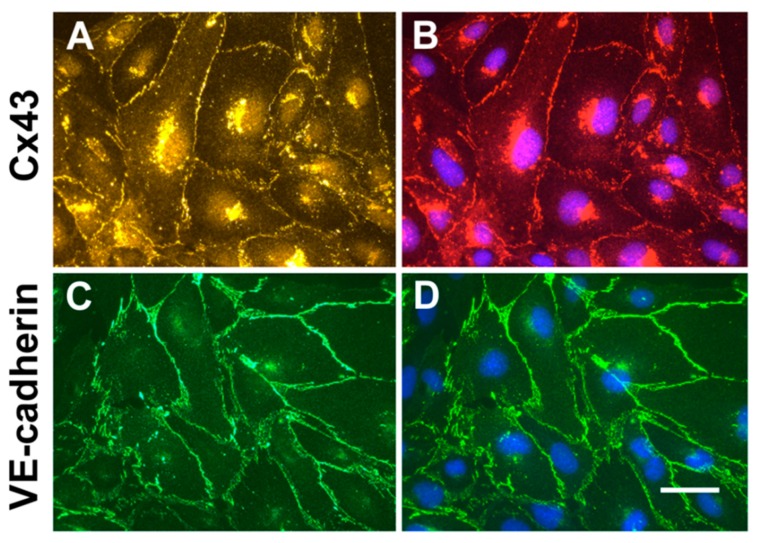
Microvascular endothelial cells have intercellular junctions containing Cx43 and VE-cadherin. (**A**,**B**) Immunolocalization of Cx43 (yellow in **A**, red in **B**). (**C**,**D**) Immunolocalization of VE-cadherin (green). Panels **B** and **D** were also stained with DAPI to visualize nuclei (blue). Bar, 10 µm.

To confirm that cultured endothelial hCMEC/D3 cells contain junctional proteins expected for endothelial cells and might be a good choice for studying secretion of extracellular vesicles and connexins, we performed immunofluorescence microscopy. Cx43 was localized to appositional membranes with a punctuate distribution (Figure 1A). Cx43 was also found within the cytoplasm; counterstaining of nuclei with DAPI (4′,6-Diamidino-2-Phenylindole, Dihydrochloride) showed that this Cx43 had a paranuclear localization (Figure 1B). The adherens junction protein, VE-cadherin was abundant in a continuous distribution at the membranes between cells, with minimal intracellular staining (Figure 1C,D).

### 2.2. hCMEC/D3 Cells Secrete Extracellular Vesicles with Characteristics of Exosomes

**Figure 2:**
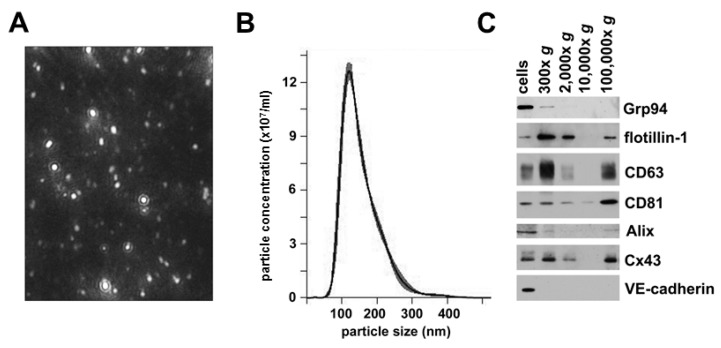
Vesicles secreted from cultured microvascular endothelial cells have properties consistent with identification as exosomes and contain Cx43. (**A**) Screen shot showing single frame of a video from the nanoparticle tracking analysis of the 100,000× *g* material. (**B**) Graph shows a representative profile of the extracellular vesicles in the 100,000× *g* pellet from the nano-tracking analysis. For this example, the mode was 120.7 nm; mean ± SD was 154 ± 54 nm; 10% of the vesicles were <98 nm; 50% were <141 nm; and 90% were <226 nm. (**C**) Immunoblots were performed on an hCMEC/D3 cell homogenate and the pellets obtained by sequential centrifugation of conditioned medium at increasing forces (300× *g*, 2000× *g*, 10,000× *g* and 100,000× *g* for the times noted in Materials and Methods). To facilitate comparisons, the gels were loaded with comparable percentages of the pellets from the sequential spins. The 100,000× *g* material contained the exosomal markers (flotillin-1, CD63, CD81 and Alix) as well as Cx43, but it did not contain detectable amounts of the endoplasmic reticulum protein Grp94 or VE-cadherin.

We used sequential centrifugation of medium from cultured hCMEC/D3 cells to isolate secreted vesicles. We performed nanoparticle tracking analysis to assess the size and homogeneity of the last 100,000× *g* fraction from this isolation protocol. This material contained a relatively homogenous population of small particles (Figure 2A). The size distribution profiles from the nanoparticle tracking analysis showed that the vesicles distributed within a rather sharp, single peak; most of the vesicles had a diameter of ~120 nm (Figure 2B). This particle size corresponds to that expected for exosomes.

To characterize further the extracellular vesicles isolated from these endothelial cells, we performed immunoblot analysis of hCMEC/D3 cell lysates and fractions from all isolation steps (Figure 2C). We probed the blots with antibodies directed against several different proteins, including the heat shock protein Grp94 (which is found in the endoplasmic reticulum) and four proteins that are often found in exosomes: flotillin-1 (a caveolar protein), two tetraspanins (CD63 and CD81), and Alix (a protein involved in endosomal trafficking). Each of these proteins was present in the hCMEC/D3 cell lysates. Grp94 was only detected in the 300× *g* fraction prepared from the culture medium; it was absent from higher speed pellets (including the final 100,000× *g* vesicles). These data suggested that the lowest speed pellet contained a substantial amount of cellular debris, but they essentially excluded an endoplasmic reticulum content or origin of the highest speed fractions. In contrast, flotillin-1, CD63, and Alix were present in the cells and the low speed pellet, absent in the 10,000× *g* fraction, and abundant in the final 100,000× *g* fraction. CD81 had low abundance in the 10,000× *g* fraction and increased abundance in the 100,000× *g* material. These immunoblots confirmed that the small extracellular vesicles contained in the pellet that required high forces (100,000× *g*) for their sedimentation contained several “exosomal marker” proteins and little contamination by cellular debris.

We also tested the extracellular vesicles derived from the hCMEC/D3 cells for the presence of lysosomal enzymes. We detected β-galactosidase, β-glucosidase, α-fucosidase, hexosaminidase, tripeptidyl-peptidase 1, palmitoyl protein thioesterase, and acidic phosphatase activities in these cells and in the 100,000× *g* fractions.

### 2.3. Extracellular Vesicles Secreted by hCMEC/D3 Cells Contain Cx43, but not VE-cadherin

We performed blots to determine the presence and relative abundance of the gap junction protein, Cx43 and the adherens junction protein, VE-cadherin in the hCMEC/D3 cell homogenates and in the fractions from their conditioned medium (Figure 2C). The Cx43 blot appeared similar to the blots for the endosomal marker proteins, showing that the connexin was abundant in the cells, the cellular debris (300× *g* and 2000× *g* pellets), and in the vesicles isolated by 100,000× *g* sedimentation. In contrast, VE-cadherin was only readily detectable in the cell lysates, suggesting that there is minimal shedding or secretion of this adherens protein. 

**Figure 3:**
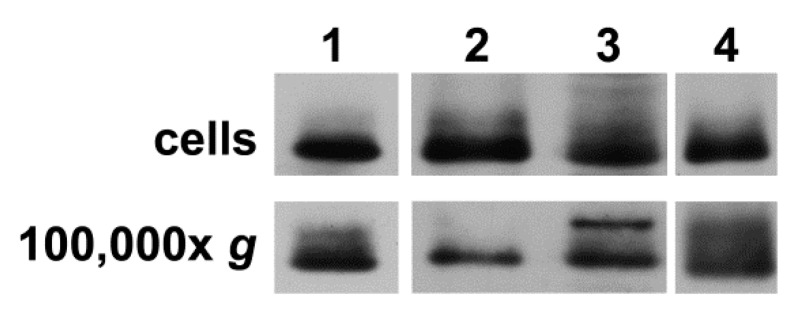
Cx43-expressing primary cells and cell lines secrete extracellular vesicles containing Cx43. Cell homogenates and the pellets from 100,000× *g* centrifugation of conditioned media were prepared as described for hCMEC/D3 cells in Materials and Methods from (1) human dermal microvascular endothelial cells, (2) human dermal fibroblasts, (3) human oligodendroglioma (HOG) cells, and (4) human glioblastoma (A172) cells. Samples were resolved by SDS-PAGE and transferred to membranes that were reacted with anti-Cx43 antibodies as described in Materials and Methods. Immunoblots show the presence of Cx43 protein in all cell homogenates and secreted vesicles.

To confirm that secretion of extracellular vesicles containing Cx43 was a general process (not restricted to this cell line), we performed similar immunoblot studies of cell homogenates and the 100,000× *g* pellets of the medium from cultures of several other cells (primary and cell lines). We found that human dermal microvascular endothelial cells, human skin fibroblasts, human oligodendroglioma (HOG) cells, and human glioblastoma (A172) cells expressed Cx43 and secreted vesicles containing Cx43 (Figure 3).

### 2.4. Staurosporine Treatment Increases hCMEC/D3 Secretion of Vesicles Containing Exosomal Proteins and Cx43

**Figure 4:**
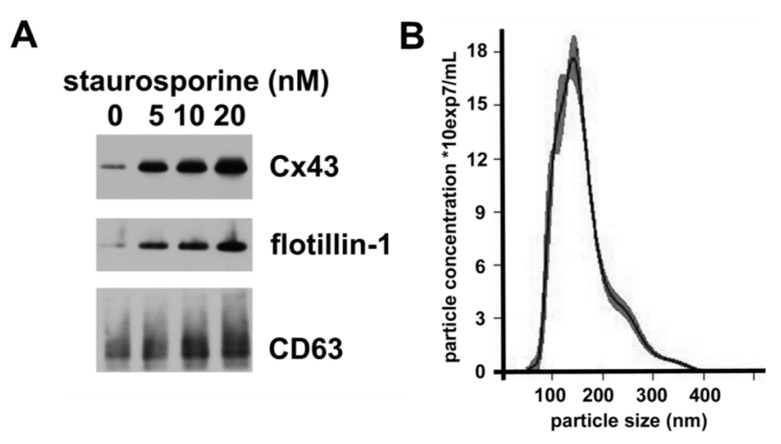
Treatment with staurosporine increases secretion of vesicles containing exosomal proteins and Cx43. (**A**) Cells were treated for 18 h with different concentrations of staurosporine (as indicated). The 100,000× *g* pellets were isolated by centrifugation of the culture medium, they were resuspended, and, for each blot, identical volumes were immunoblotted to detect Cx43, flotillin-1 and CD63. (**B**) Graph shows the profile from the nanoparticle tracking analysis of the extracellular vesicles in the 100,000× *g* pellet prepared from cells treated with 10 nM staurosporine. For this profile, the mode was 141.6 nm; mean ± SD was 161 ± 57 nm; 10% were <101 nm; 50% were <148 nm; and 90% were <241 nm.

The release and properties of exosomes can be affected by various cellular stresses. To test whether the secretion of Cx43-containing vesicles could be modulated, we treated hCMEC/D3 cells with various doses of the apoptosis inducer, staurosporine for 18 h; we observed no toxicity of the lowest staurosporine doses, but we saw some cell death at 20 nM. We isolated the extracellular vesicles by the sequential centrifugation protocol, and we studied them by immunoblotting and nanoparticle tracking analysis. Immunoblotting of comparable percentages of the high-speed pellets showed that increasing concentrations of staurosporine were associated with increased levels of flotillin-1 and CD63, suggesting the release of more vesicles by these cells (Figure 4A). Increased amounts of staurosporine also caused increased abundance of vesicular Cx43. Nanoparticle analysis showed that these vesicles were predominantly small, but there was some increased abundance of particles on the larger side of the curve (Figure 4B).

## 3. Discussion

Our data presented in this manuscript confirm that the gap junction protein, Cx43, is secreted in extracellular vesicles by the endothelial cell line hCMEC/D3. This secretion seems specific, since another intercellular junction protein, VE-cadherin (a component of adherens junctions) was not present in these extracellular vesicles.

Many of the Cx43-containing extracellular vesicles secreted by our cells correspond to the class of small extracellular vesicles that have frequently been called exosomes [15]. In untreated cells, they constitute a rather uniformly sized population with a diameter just larger than 100 nm. They contain several proteins typical of exosomes (flotillin-1, CD63, CD81 and Alix), while an endoplasmic reticulum-resident protein (Grp94) is absent. It is unlikely that the presence of Cx43 in the small extracellular vesicles isolated from hCMEC/D3 cells is an artifact of our isolation method, since we also detected Cx43 by immunoblotting of “exosomes” isolated from hCMEC/D3 conditioned medium using a precipitation kit (total exosome isolation kit, Thermo Fisher Scientific Inc., Waltham, MA, USA). We are confident that the presence of Cx43 in small extracellular vesicles is not a phenomenon unique to hCMEC/D3 cells, since it was found in similar vesicles of other sources by Soares et al. [12], and we have found it in vesicles derived from other endothelial cells, glioblastoma cells, oligodendroglioma cells, and fibroblasts (Figure 3). 

Interestingly, we found that the vesicles secreted by the hCMEC/D3 cells contained lysosomal enzyme activities. This is consistent with some prior studies that have shown the presence of lysosomal proteins in isolated exosomes, including cathepsin D and LAMP-1 in exosomes from Alzheimer disease patients [16] and glucocerebrosidase in exosomes from Parkinson’s disease patients [17]. Hopwood and associates [18] have found that a lysosomal enzyme which normally degrades heparin sulfate is present in extracellular vesicles isolated from HEK293T conditioned media or from urine; the enzyme co-immunoprecipitates with Alix and is likely transported by these vesicles.

The stimulated secretion of extracellular vesicles containing Cx43 following staurosporine treatment is likely a complex, multifactorial process. In a variety of cells (including endothelial cells), stimulation of apoptosis stimulates release of vesicles [19,20]. The small size and the presence of various markers suggest that many of these are small extracellular vesicles (exosomes). However, endothelial cells secrete a variety of vesicles of different sizes, including exosomes (≤ 100 nm), microparticles (0.1–1.0 µm), and apoptotic bodies (≥ 1 µm) [21,22]. The presence of vesicles with larger diameters in the staurosporine-treated cells may reflect secretion of some small microparticles in addition to exosomes. Previous studies have shown that endothelial cells secrete microparticles with different contents in response to apoptotic as compared to inflammatory stimuli [20]. The vesicles secreted by our hCMEC/D3 cells are too small to represent apoptotic bodies.

Our data and recent publications from other laboratories have found Cx43 in small extracellular vesicles or exosomes. However, it is not certain why this gap junction protein is present in these vesicles nor what function(s) it might serve. There are several potential explanations:

(1) The vesicular connexin could represent cellular “garbage” shed from cells. Clearly, culture medium contains connexin in cellular/membrane material (300× *g* pellet). This possibility is also supported by the presence of lysosomal enzymes, suggesting the connexin might be derived from material in the degradative pathway. However, we suspect that the cultured cells perform some specific vesicular secretion of Cx43, since the exosome-like vesicles (in the 100,000× *g* fraction) did not contain an ER protein or another junction protein. Moreover, Soares et al. [13] found Cx43 in small extracellular vesicles isolated from patient blood, substantiating in vivo secretion. 

(2) The exosomal Cx43 might facilitate interaction of small extracellular vesicles with their target cells. Soares et al. [13] have presented data suggesting that exosomal Cx43 formed channels connecting the vesicle to the recipient cell, and it facilitated transfer of signaling molecules based on their observation of DNA and luciferin passage. While we have not studied this process, we suspect that the abundance and function of such channels might be rather low. Rather, Cx43 might be more important for the interaction/fusion of vesicle and target cell. This possibility is supported by the studies of exosome-like “connectosomes”: cell-derived lipid vesicles that contain functional gap junction channels and encapsulate molecular cargos such as chemotherapeutic drugs [23,24]. 

(3) The connexin might have a structural/organizational role in the vesicle. It has been suggested that certain connexin domains may recruit and bind RNA [25]. 

(4) It is also possible that Cx43 is important as part of the vesicular cargo because of its effects once released into the target cell. A variety of recent studies have shown that domains within the Cx43 protein can traffic to the nucleus and modulate gene transcription [26,27]. Through this kind of process, a few Cx43 molecules might have a substantial effect on the recipient cells.

## 4. Materials and Methods 

### 4.1. Cell Culture

Immortalized human brain endothelial cells (hCMEC/D3) were purchased from CELLutions Biosystems Inc. (Burlington, Ontario, Canada). Cells were plated on 100 mm tissue culture dishes coated with rat collagen at 150 µg/mL (Thermo Fisher Scientific Inc., Waltham, MA, USA). Culture medium consisted of: EBM-2 medium (Lonza Walkersville, Inc.; Allendale, NJ, USA) supplemented with 5% fetal bovine serum (FBS, Serum Source International, Charlotte, NC, USA); 1% penicillin/streptomycin, 5% chemically defined lipid concentrate, 10 mM 4-(2-hydroxyethyl)-1-piperazineethanesulfonic acid (HEPES), basic fibroblast growth factor (bFGF at 1 ng/mL) (Thermo Fisher Scientific Inc.); 1.4 µM hydrocortisone, ascorbic acid (5 µg/mL) (SIGMA-Aldrich, Saint Louis, MO, USA).

Human dermal microvascular endothelial cells (CC-2543) were purchased from Lonza Walkersville, Inc.) and maintained according to manufacturer’s instructions in endothelial growth medium (EGM-2MV Bullet Kit; Lonza Walkersville) supplemented with 5% fetal bovine serum. At passage 10, cells were split for the experiment. Human skin fibroblasts were grown in RPMI (Roswell Park Memorial Institute) medium (Thermo Fisher Scientific Inc.) supplemented with 10% fetal bovine serum and 1% penicillin/streptomycin. The human oligodendroglioma cell line, HOG, was established as described earlier [28,29]. The human glioblastoma cell line, A172, was obtained from ATCC (CRL-1620). HOG and A172 cells were maintained in Dulbecco’s Modified Eagle Medium supplemented with 10% fetal bovine serum and 1% penicillin/streptomycin. 

All cells were incubated in cell culture incubators at 37 °C in an atmosphere containing 5% CO_2_.

### 4.2. Isolation of Extracellular Vesicles 

Extracellular vesicles were isolated from conditioned medium. Cells were incubated in growth medium deprived of FBS for 18 h prior to vesicle isolation. Then, medium was collected and subjected to differential centrifugation at 4 °C, starting with centrifugation at 300× *g* for 5 min (Sorvall Legend RT tabletop centrifuge), followed by 2 000× *g* for 20 min (Sorvall Legend RT tabletop centrifuge), 10,000× *g* for 30 min (Sorvall RC6 Plus Centrifuge, SS34 Rotor), and finally, 100,000× *g* for 18 h (Beckman L8-70M ultracentrifuge, SW55 Ti rotor). Pellets from all steps of purification were resuspended in 200 µL of phosphate buffered saline (PBS) containing 50 mM sodium fluoride, 0.5 mM sodium orthovanadate, and mini EDTA-free protease inhibitors (SIGMA-Aldrich) (one tablet per 5 mL of PBS). 

### 4.3. Nanoparticle Tracking Analysis

Nanoparticle tracking analysis (NTA) was performed using the Nanosight NS300 (Malvern Panalytical Inc., Westborough, MA, USA). Isolated extracellular vesicles were diluted 1:30 with PBS and injected into the 405 nm laser chamber with a constant output controlled by a syringe pump. Three recordings were performed for each sample. NTA software was used to determine the size of vesicles. 

### 4.4. Antibodies

Primary antibodies used for immunoblotting included: anti-Grp94 (sc-393402, Santa Cruz, Biotechnology, Inc., Dallas, TX, USA) mouse monoclonal antibodies raised against amino acids 200-411 of human Grp94 used at 1:200 dilution; anti-flotillin-1 (sc-133153, Santa Cruz, Biotechnology, Inc.) mouse monoclonal antibodies raised against amino acids 324-427 of human flotillin-1 used at 1:200 dilution; anti-CD63 (sc-5275, Santa Cruz, Biotechnology, Inc.) mouse monoclonal antibodies raised against full length human CD63 at 1:500 dilution; mouse monoclonal anti-CD81 (sc-7637, Santa Cruz, Biotechnology, Inc.) at 1:100 dilution; mouse monoclonal anti-Alix (sc-53538, Santa Cruz, Biotechnology, Inc.) raised against full length human Alix used at 1:500. Cx43 was detected using rabbit polyclonal antibodies directed against amino acids 363-382 of human/rat Cx43 (C6219, SIGMA-Aldrich) at 1:5000 dilution for immunoblotting and 1:250 for immunofluorescence. VE-cadherin was detected using mouse monoclonal antibodies (sc-9989, Santa Cruz, Biotechnology, Inc.) specific for an epitope between amino acids 768-784 of human VE-cadherin used at 1:2000 dilution for immunoblotting and at 1:100 for immunofluorescence.

Cy3-conjugated goat anti-rabbit IgG; AlexaFluor 488 goat anti-mouse IgG and horseradish peroxidase (HRP)-conjugated goat anti-rabbit or anti-mouse IgG antibodies were obtained from Jackson ImmunoResearch (West Grove, PA, USA).

### 4.5. Immunofluorescent Staining

For immunofluorescence staining, cells were grown on cover slips in 12 well tissue culture plates. When cells reached confluency, they were fixed with 4% (*w*/*v*) paraformaldehyde in PBS for 15 min at room temperature and then washed with PBS. The cell membranes were permeabilized by incubation with PBS containing 0.1% Triton X-100 for 10 min, then blocked twice with 10% heat-inactivated normal goat serum in PBS containing 0.1% Triton X-100 for 30 min at room temperature. Cells were incubated with primary antibodies diluted in blocking solution overnight at 4 °C. Following incubation with primary antibodies, cover slips were washed 3 times for 5 min with PBS. Then they were incubated with secondary reagents at room temperature for 1 h and subsequently extensively washed with PBS. For nuclear counterstaining, cover slips were incubated for 15 min with DAPI (Thermo Fisher Scientific Inc.) at a concentration of 500 µg/mL and again extensively rinsed with PBS. Each cover slip was mounted on glass slide using Prolong Gold anti-fade reagent (Thermo Fisher Scientific Inc.). Slides were sealed and stored in darkness at 4 °C. Cells were studied using the 40× Plan Apochromat objective in an Axioplan 2 microscope (Carl Zeiss Meditec, Munich, Germany). Images were captured with a Zeiss Axiocam digital camera using Zeiss AxioVision software.

### 4.6. Immunoblot Analysis 

Cell homogenates were prepared as described by Gong et al. [30]. Immunoblots were performed essentially as described in our previous studies [31]. The protein concentrations of homogenates were determined using the method of Bradford [32] (Bio-Rad, Richmond, CA, USA). Aliquots of cell homogenates containing 100 ng–50 µg of protein or 1–30 µL of extracellular vesicles were separated by SDS-PAGE on gels containing 8% acrylamide (for VE-cadherin), 14% (for CD63 and CD81) or 10% (for all other protein targets). Polyacrylamide gels were run under reducing conditions or non-reducing conditions (for CD63, CD81 and VE-cadherin). Proteins were blotted onto Immobilon-P membranes (SIGMA-Aldrich). ProSieve Protein Colored Markers standards (Lonza Walkersville, Inc.) were used to calibrate the gels. For most targets, immunoblots were developed using enhanced chemiluminescence reagents (ECL Prime, GE Healthcare Biosciences, Pittsburgh, PA, USA); Alix and CD81 blots were reacted with SuperSignal™ West Femto Maximum Sensitivity Substrate (Thermo Fisher Scientific Inc.). Chemiluminescence was detected by exposing blots to X-ray film. 

### 4.7. Cell Treatments

hCMEC/D3 cells were treated with 0, 5, 10 or 20 nM staurosporine in dimethyl sulfoxide (DMSO; SIGMA-Aldrich) for 18 h. The concentration of DMSO in all samples was adjusted to 0.12%. At the end of treatments, cells were harvested for immunoblot analysis. 

## 5. Conclusions

Many studies have established that extracellular vesicles are shed by tumor and endothelial cells, and they may have physiological, pathologic, diagnostic, and therapeutic roles. Our confirmation that endothelial-derived small extracellular vesicles contain exosomal proteins and Cx43 should stimulate further cancer related research in this area.
